# A Rare Case of Secondary Bacterial Peritonitis from *Clostridium perfringens* in an Adult Patient with Noncirrhotic Ascites and a Krukenberg Tumor: Report of a Case

**DOI:** 10.1155/2011/705816

**Published:** 2011-07-09

**Authors:** Scott R. Kelley, George M. Kerlakian

**Affiliations:** Department of Surgery, Hatton Research Institute, Good Samaritan Hospital, 375 Dixmyth Avenue, Cincinnati, OH 45220, USA

## Abstract

Secondary bacterial peritonitis, in comparison to spontaneous, presents with a surgically treatable intraabdominal source for infection such as a gastrointestinal perforation or abscess and is nearly always polymicrobial. We present a rare case of secondary bacterial peritonitis from *Clostridium perfringens* in an adult patient with noncirrhotic ascites and a Krukenberg tumor.

## 1. Introduction

Secondary bacterial peritonitis presents with a surgically treatable intraabdominal source for infection, such as a gastrointestinal perforation or abscess, and is nearly always polymicrobial [1]. With a lack of gastrointestinal perforation, mono-organismal intraabdominal anaerobic bacteria are rarely identified. This is secondary to the high oxygen tension of ascitic fluid, the intestinal wall, and surrounding tissues as well as the relative inability of anaerobes to translocate across intestinal mucosa [2]. We present a rare case of secondary bacterial peritonitis from *Clostridium perfringens* in an adult patient with noncirrhotic ascites and a Krukenberg tumor.

## 2. Case Report

A 51-year-old white female with a known history of metastatic gastric cancer receiving chemotherapy (5-FU, Oxaliplatin, Leucovorin) presented with a 12–24 hour history of nausea, bilious emesis, trivial hematemesis, mild abdominal pain, and distention. On presentation, she was lethargic, hypoxic, hypotensive, and minimally responsive though alert and oriented. Her last dose of chemotherapy was three days prior. Laboratory evaluation revealed a WBC of 1.5 with 80% bands, creatinine 2.13, ABG 7.31/34/53/16/−9/85%, lactic acid 9.9, CvO^2^ 41, albumin 1.8, prealbumin 8.5, transferrin 85, International normalized ration (INR) 1.7, partial thromboplastin time (PTT) 56.2, and normal liver function test other than an elevated alkaline phosphatase level of 284. 

Other than metastatic gastric cancer, her medical history was only significant for biliary and right ureteral obstruction (secondary to metastasis), which necessitated prior placement (over a year preceding her presentation) of biliary and ureteral stents for decompression. She had no surgical history, was on Lexapro, Nystatin, and her chemotherapy regimen, and was without allergies. She did not smoke or drink and had no family history.

**Figure 1:**
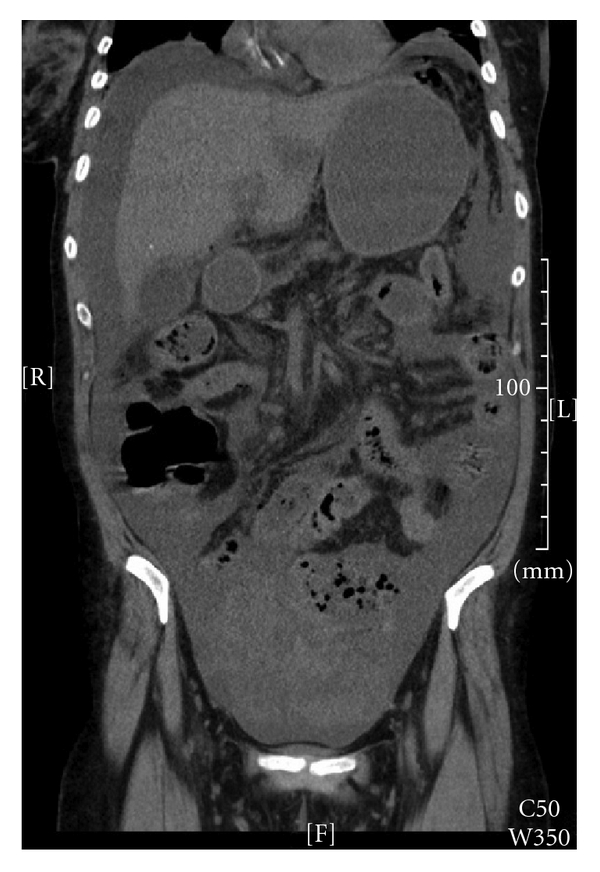
CT showing mass-like cystic structure in pelvis with air bubbles.

The patient was afebrile with a heart rate of 119, blood pressure of 77/47, and respiratory rate of 29 with a SaO^2^ of 94% on a 100% nonrebreather. She appeared cachetic and in distress. Her abdomen was distended, firm, minimally tender without overt signs of peritonitis, and an upright CXR revealed free air under both hemidiaphragms. After aggressive fluid resuscitation, which led to improvement in her vital signs, an abdominal and pelvic computed tomography scan was obtained, exposing significant intra-abdominal ascites and a large nonhomogenous cystic mass-like structure in the pelvis surrounded by numerous air bubbles, along with scattered free air throughout the peritoneal cavity (Figure 1). 

Exploratory laparotomy revealed a large hemorrhagic, necrotic appearing cystic mass in the rightward pelvis. Over two liters of cloudy nonmalodorous ascitic fluid was aspirated on entry into the abdominal cavity. The pelvic mass, which was removed along with the right fallopian tube, was intimately involved with the right ovary, sparing surrounding structures. The left ovary was normal in appearance. No other etiology was appreciated after extensive intraoperative exploration. Pathology revealed a 15.9 × 12.5 × 6.2 cm poorly differentiated adenocarcinoma consistent with metastatic gastric cancer, and fluid and tissue cultures were positive for *Clostridium perfringens*. The ascitic fluid was not examined for neoplastic cells. Blood cultures obtained during her initial evaluation also grew* Clostridium perfringens*. After a prolonged hospital course and treatment for *C. perfringens,* she was discharged home in good condition.

## 3. Discussion

*Clostridium perfringens* is a Gram-positive, anaerobic, spore-forming, gas-producing bacilli found in rich cultivated soil, with vegetative forms found on the skin as well as in the vagina and colon. Though considered anaerobic, *C. perfringens* is cable of growth in 30% oxygen tension, allowing the organism to freely proliferate. With the capability to produce over 17 different exotoxins, the host inflammatory response is characteristically, though anomalously, very low. Presenting symptoms are frequently not associated with peritonitis even in the face of infected ascites and free perforation, and though not completely understood, exotoxins are hypothesized to play a significant role. Theta toxin causes leukocyte degeneration and polymorphonuclear cell destruction, which may explain the paucity of host inflammatory response. It is also known to induce direct vascular injury, hemolysis, and cytolysis. Alpha toxin, the most notable and common, causes lysis of leukocytes, red blood cells, platelets, fibroblasts, and myocytes. Others such as kappa toxin facilitate the rapid spread of necrosis through tissues by destroying connective tissue. 

Isolation of *Clostridium perfringens* as a cause of spontaneous bacterial peritonitis (SBP) is an extremely rare, though known, event with only a mere handful of cases reported in the literature [3–7]. Most cases of SBP are detailed in patients with cirrhosis, especially Child-Pugh class C, whom have ascites, and greater than 60% of cases are secondary to Gram-negative enteric bacteria (*Escherichia coli* and *Klebsiella-pneumoniae*). Isolation of aerobic organisms is seen in roughly 25% of cases, with *streptococci* and *enterococci* being the most frequently isolated species [8]. Secondary bacterial peritonitis, in comparison to spontaneous, is predominately polymicrobial and presents with a surgically identifiable cause. Early diagnosis and surgical intervention is necessary, and if merely treated with antibiotics, mortality rates approach one-hundred percent [9]. 

In the face of a nonsurgically identifiable cause of bacterial peritonitis (spontaneous), intestinal bacterial overgrowth, intestinal permeability, bacterial translocation, and alterations in the immune, reticuloendothelial, and ascitic fluid defense systems are all hypothesized to play a part [2]. Intestinal bacterial overgrowth, seen in patients with advanced cirrhosis as a result of portal hypertension, has been noted to be a prerequisite for the facilitation of bacterial translocation. Increased intestinal permeability results from vascular congestion, edema, and increased interepithelial cell spacing, which is also the result of portal hypertension and is another prerequisite for translocation. Bacterial translocation allows microorganisms to directly colonize mesenteric lymph nodes and spread both hematogenously as well as to surrounding ascitic fluid though without overgrowth and increased permeability, translocation is unlikely to occur. Alterations in the humoral or cellular mediated immunity can also greatly increase the risk of peritoneal infections. The bactericidal activity present in ascitic fluid, mainly through the complement cascade system, provides patients with adequate complement levels as defense from developing ascitic bacterial infections [10]. 

Our particular patient presented with a nonperitoneal examination. Given her computed tomography findings of free air and a pelvic mass, the decision to proceed with exploratory laparotomy to search for a gastrointestinal perforation was made. The intraperitoneal air was a perplexing phenomenon until the final tissue and fluid cultures revealed *Clostridium perfringens*. Though the right ovary appeared to be the source for the *C. perfringens*, this cannot be concluded with absolute certainty. We hypothesize that with her severe protein calorie malnutrition, ascites, and altered humoral and cellular mediated immunity as a result of her chemotherapy, vaginal clostridial translocation likely occurred, migrating to her ovarian tumor, which spread to her ascitic fluid and bloodstream. With her lack of cirrhosis and portal hypertension, intestinal bacterial overgrowth, increased permeability, and subsequent intestinal translocation are less likely causes of her mono-organismal infection. Though biliary tract origin and stenting are known phenomena for causing *C. perfringens* bacterial peritonitis, our patient was stented well over a year prior to her presentation and her stent had not been manipulated, making this a less likely source in our particular patient though possible. In conclusion, when evaluating a patient with abdominal free air, surgical consultation should be entertained, as surgical intervention may be necessary.
